# Identification of Tumor Budding-Associated Genes in Breast Cancer through Transcriptomic Profiling and Network Diffusion Analysis

**DOI:** 10.3390/biom14080896

**Published:** 2024-07-24

**Authors:** Panisa Janyasupab, Kodchanan Singhanat, Malee Warnnissorn, Peti Thuwajit, Apichat Suratanee, Kitiporn Plaimas, Chanitra Thuwajit

**Affiliations:** 1 Advance Virtual and Intelligent Computing (AVIC) Center, Department of Mathematics and Computer Science, Faculty of Science, Chulalongkorn University, Bangkok 10330, Thailand; panisa.janyasupab@gmail.com; 2 Department of Immunology, Faculty of Medicine Siriraj Hospital, Mahidol University, Bangkok 10700, Thailand; ksinghanat23@gmail.com (K.S.); peti.thu@mahidol.edu (P.T.); 3 Department of Pathology, Faculty of Medicine Siriraj Hospital, Mahidol University, Bangkok 10700, Thailand; maleewarn@yahoo.com; 4 Department of Mathematics, Faculty of Applied Science, King Mongkut’s University of Technology North Bangkok, Bangkok 10800, Thailand; apichat.s@sci.kmutnb.ac.th; 5 Intelligent and Nonlinear Dynamics Innovations Research Center, Science and Technology Research Institute, King Mongkut’s University of Technology North Bangkok, Bangkok 10800, Thailand

**Keywords:** breast cancer, differential expression analysis, mutual information, network diffusion, tumor budding

## Abstract

Breast cancer has the highest diagnosis rate among all cancers. Tumor budding (TB) is recognized as a recent prognostic marker. Identifying genes specific to high-TB samples is crucial for hindering tumor progression and metastasis. In this study, we utilized an RNA sequencing technique, called TempO-Seq, to profile transcriptomic data from breast cancer samples, aiming to identify biomarkers for high-TB cases. Through differential expression analysis and mutual information, we identified seven genes (*NOL4*, *STAR*, *C8G*, *NEIL1*, *SLC46A3*, *FRMD6*, and *SCARF2*) that are potential biomarkers in breast cancer. To gain more relevant proteins, further investigation based on a protein–protein interaction network and the network diffusion technique revealed enrichment in the Hippo signaling and Wnt signaling pathways, promoting tumor initiation, invasion, and metastasis in several cancer types. In conclusion, these novel genes, recognized as overexpressed in high-TB samples, along with their associated pathways, offer promising therapeutic targets, thus advancing treatment and diagnosis for breast cancer.

## 1. Introduction

Breast cancer is the most commonly diagnosed cancer, with an estimated 2.3 million estimated new cases and 684,996 deaths in 2020 [1]. Tumor budding (TB), defined as an isolated single cell or a cluster of up to four cancer cells located at the invasive tumor front [2], has been identified as having prognostic significance in breast cancer [3]. Identifying genes differentially expressed between high and low TB levels can be proposed as theragnostic markers for better management of breast cancer.

Nowadays, gene expression datasets have proliferated greatly due to technological advancements. There are several techniques for obtaining gene expression data, such as microarray and RNA sequencing. Recently, Templated Oligo-Sequencing (TempO-Seq) has been introduced as a new transcriptomic platform that combines the advantages of microarray and RNA sequencing technology [4]. TempO-Seq is a high-throughput method for profiling gene expression directly from tissue sample lysates, including FFPE tissues, without needing RNA extraction [5,6]. Its advantage lies in its ability to provide quantitative measurements of gene expression with high sensitivity and precision [7]. TempO-Seq provides count data for individual genes and is employed for comprehensive whole transcriptome profiling, primarily focusing on toxicogenomic research [7]. The standard pipeline for differential expression analysis, especially DESeq2, is well suited to TempO-Seq as well. The effectiveness of different normalization methods was evaluated when comparing two classes of TempO-Seq data, revealing that DESeq2 can provide relatively accurate results for absolute fold change levels of 2.0 or greater [8]. However, clinical data extracted from breast cancer are normally limited, with small numbers of cases found. A small and imbalanced set of samples can be problematic when analyzing differential gene expression because it limits the statistical power and robustness of the analysis. In the context of small sample sizes, mutual information may be more robust than traditional statistical tests because it does not rely on assumptions about the underlying distribution of the data and can handle non-linear relationships effectively. This makes it particularly useful for identifying subtle but informative features or genes that may be missed using methods based solely on mean differences or variance [9]. Despite having been a classical method for a long time, mutual information has demonstrated robustness [10] and effectiveness in handling imbalanced data [11,12], making it applicable across various domains. For instance, mutual information was employed alongside an improved Lasso method to filter unrelated genes and eliminate redundant ones, thus selecting informative genes from microarray gene expression data [13]. The large dimensionality of microarray data is reduced through combining mutual information and the Bayes theorem [14]. Additionally, biomarkers for stomach adenocarcinoma have been successfully identified through a combination of the “limma” method and joint mutual information [15].

Currently, it is evident that to enhance our comprehension of the functional pathways associated with biomarkers of interest, the utilization of network diffusion methods has proven effective in the discovery of additional relevant genes related to these biomarkers [16,17,18,19]. Consequently, this approach yields a more confident set of genes pertinent to biomarkers, thereby facilitating more robust pathway enrichment analyses that yield biologically meaningful results. Therefore, we then employed TempO-Seq technology to extract the transcriptomic data and to identify biomarkers associated with TB levels in breast cancer. Both differential expression analysis and mutual information were utilized to screen for novel biomarkers of high TB. Functional pathways relevant to the markers obtained were also retrieved. Literature support and evidence for further investigation into the diagnosis and prognosis for breast cancer were then discussed.

## 2. Materials and Methods

### 2.1. The Study Workflow

**Figure 1:**
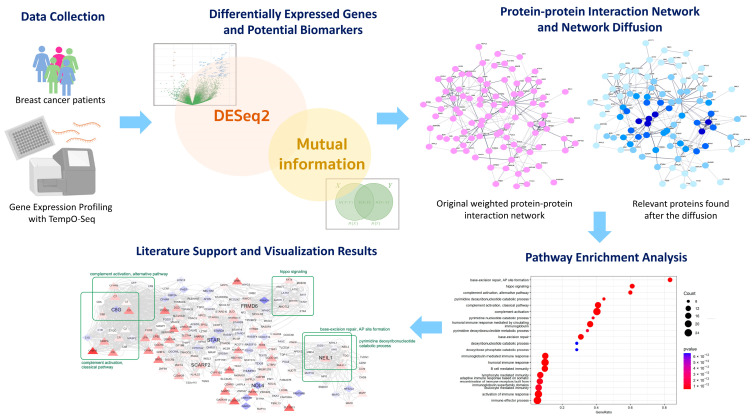
The workflow for identifying potential biomarkers of high-TB breast cancer and their related functional pathways.

The workflow of our experiment and gene expression analysis is summarized in Figure 1. Initially, the transcriptomic data were processed using TempO-Seq, including preprocessing, correction, and normalization, until read counts were obtained for all genes. Subsequently, DESeq2 identified differentially expressed genes (DEGs) between high- and low-TB samples. Additionally, mutual information was employed to select genes with clearly distinguishable expression levels for high or low budding levels. The overlapping genes from these methods were identified as potential biomarkers for high-TB cases. Furthermore, a protein–protein interaction network and a network diffusion technique were applied to searching for more relevant proteins associated with TB progression, using these biomarkers as seeds. Finally, biological pathway enrichment was conducted to gain insights into the mechanisms underlying these biomarkers and their interactions within the protein–protein interaction network.

### 2.2. Breast Cancer Sample Collection, Transcriptomic Data Preparation, and Tumor Budding Evaluation

Fifteen formalin-fixed paraffin-embedded (FFPE) breast cancer tissues from breast cancer patients who underwent surgery at Faculty of Medicine Siriraj Hospital, Mahidol University, between 2004 and 2017 were utilized for the study. Clinicopathological data, including patient age at diagnosis, histological subtype, tumor size, lymph node status, tumor staging, and survival status (measured by overall survival, OS), were collected (see Table 1). The protocol for collecting the samples and the clinicopathological data collection was approved by the Siriraj Institution Review Board according to human ethics standards (COA no. si542/2022). The cancer cell areas in the FFPE samples were labeled by a pathologist and sent for transcriptomic analysis using Templated Oligo-Sequencing (TempO-Seq) analysis (BioClavis, Glasgow, UK). Briefly, the FFPE tissues were deparaffinized and then digested. The tissue lysate was incubated with detector oligos annealing to the targeted RNA template [7]. Amplification of the ligated oligos was performed using a unique primer set for each sample, introducing a sample-specific barcode and Illumina adaptors. The samples were then sequenced on an Illumina HiSeq 2500 High Output v4 flow cell and analyzed using BCL2FASTQ software v2.20 (Illumina, San Diego, CA, USA). The obtained FASTQ files were aligned with the Human Whole Transcriptome v2.0 panel, consisting of 22,537 probes, using STAR [20]. The gene expression profile contains 15 cases, including 12 high- and 3 low-TB samples. Our gene expression dataset and its associated FASTQ files can be found in the NCBI Gene Expression Omnibus [21], with accession number GSE262825. Initially, the maximum mean gene expression was selected in case of duplication, resulting in a reduction in the total 22,537 probes to 19,703 genes.

For tumor budding evaluation, the FFPE slides were sent to the Department of Pathology, Faculty of Medicine Siriraj Hospital, Mahidol University, for cytokeratin staining using routine services. Briefly, 4-µm-thickness FFPE sections were incubated overnight at 4 °C in a humidified chamber with a 1:350 dilution of anti-pan-cytokeratin (AE1/AE3, Dako). Subsequently, the sections were processed using the K8002 detection kit, which includes an anti-mouse EnVision+ System HRP-labeled polymer (K4001), a substrate buffer, and diaminobenzidine (DAB) solution (Dako). The slides were then counterstained with hematoxylin. The stained slides were scanned at a resolution of 0.12 microns/pixel using a 3DHISTECH PANNORAMIC 1000 microscope by 3DHISTECH Ltd. and CaseViewer/QuantCenter software 2.4.0. (Sysmex, Budapest, Hungary), with a 40× objective lens, and saved in NRXS format. Quantification of positive membranous staining for pan-cytokeratin was performed using the image analysis software CaseViewer version 2.4 (3DHISTECH Ltd., Budapest, Hungary).

Tumor budding was defined as either one isolated tumor cell or up to four cells in clusters detached from the main tumor mass [22]. Within a low-power field (x50 original magnification), five areas with the highest budding number at the invasive margins were selected. Within a high-power field (200× original magnification), the number of budding cells was counted. According to the International Tumor Budding Conference (ITBCC) guidelines, the number of buds was categorized as low (0–4 buds), intermediate (5–9 buds), and high (≥10 buds). The tumor budding counts were independently reviewed by two examiners, one of whom was a medical doctor.

All the methods described in this study were performed in accordance with the relevant guidelines and regulations set forth by Siriraj Institutional Review Board (SIRB Protocol No.: 277/2565(IRB4), COA no. Si 542/2022), ensuring full compliance with the international guidelines for human research protection, such as the Declaration of Helsinki, the Belmont Report, CIOMS Guidelines, and the International Conference on Harmonization in Good Clinical Practice (ICH-GCP). Clinical samples from the patients were obtained after acquiring informed consent from the patients and were archival specimens which covered their usage in accordance with the protocol approved by Siriraj Institutional Review Board.

### 2.3. Differential Expression Analysis

Differential expression analysis is conducted using the “DESeq2” package in R [23]. The first step involves count normalization, which is performed to facilitate comparisons between samples. Next, the model estimates gene-wise dispersions to generate more accurate estimates of dispersion. Subsequently, the negative binomial model is fitted, and the Wald test is conducted to test the hypothesis. Finally, statistical information is prepared, including the log fold change (log*FC*) and *p*-value. log*FC* can be computed according to $logFC={log}_{2}\left(\frac{x_{highTB}}{x_{lowTB}}\right)$, where $x_{highTB}$ and $x_{lowTB}$ are the normalized counts of high- and low-TB samples, respectively. Positive and negative log*FC* values occur when a gene is up-regulated and down-regulated, respectively. Next, the *p*-value from DESeq2 tests the null hypothesis that there is no differential expression across the two groups. If the mean expression levels of the two groups are significantly different, the *p*-value of the gene is smaller. The adjusted *p*-value (*adj.p.val*) is then computed using the Benjamini– Hochberg method. Following the standard procedure, we select a subset of genes with the condition that *adj.p.val* < 0.05 for DEGs.

### 2.4. Mutual Information (MI)

The idea behind mutual information is to measure the relationship between two random variables or quantify the amount of information obtained from one random variable by observing another random variable. Let X and Y be random variables. MI quantifies the information shared between *X* and *Y* and is evaluated by MI(X,Y)=H(X)+H(Y)−H(X,Y), where $H\left(X\right)=-\sum_{x\in X}p\left(x\right)log(p\left(x\right))$, which is the entropy or the expected uncertainty in a random variable *X*, and $H\left(X,Y\right)=-\sum_{x\in X}\sum_{y\in Y}p\left(x,y\right)log(p\left(x,y\right))$ is the joint entropy, which measures the uncertainty when considering two random variables simultaneously. $p\left(x\right)$ and $p\left(y\right)$ stand for the marginal probability functions of *X* and Y, respectively, while $p\left(x,y\right)$ refers to the joint probability of both variables occurring together. In this study, the “praznik” [24] package in R is utilized, where *X* represents the normalized count of each gene and Y is a binary class consisting of high- and low-TB samples. In the scenario of scoring genes, $H\left(Y\right)$ is equal for all genes. Therefore, a high entropy of *X*, $H\left(X\right),$ indicates substantial unpredictability in the gene expression across the samples. Similarly, a high joint entropy $H\left(X,Y\right)$ signifies considerable variability in the combined distribution of gene expression and class labels. The MI score ranges from 0 to infinity. A mutual information score of 0 implies that knowing one variable provides no information about the other. Conversely, a high MI value suggests a strong relationship or dependency between variables X and Y. In the context of genes and classes, a gene with a higher MI score implies that it gains more information when the class is known. Therefore, when selecting genes based on mutual information scores, the set of genes with the highest MI scores is chosen. These genes are considered to have the strongest association with the class variable, making them potentially more informative for tasks such as classification or understanding the relationship between gene expression patterns and class labels.

### 2.5. The Protein–Protein Interaction Network and Network Diffusion Technique

The protein–protein interaction network was constructed from STRING database version 12.0 [25]. A subnetwork is selected based on the criterion of a combined score greater than 0.4, resulting in 19,038 protein nodes and 901,089 edges. Network diffusion is a network-based technique that requires the input of a network with initial seed nodes. Let *G* be the network with *n* nodes, and *W* be the weighted adjacency matrix of *G*. Assume that *D* is an *n* × *n* diagonal matrix where $D_{ii}=\sum_{j}W_{ij}$, and L=D−W is called the Laplacian of the graph. The regularized Laplacian kernel is introduced in [26] as $K={\left(I+\alpha L\right)}^{-1}$, where *I* is an *n* × *n* identity matrix. The network diffusion score is computed as $S_{diff}=K\cdot y$, where *y* is a binary vector of length *n*, with 1 assigned to the seed nodes and 0 otherwise. The overlapping set of genes found by the DESeq2 and mutual information techniques was identified as potential biomarkers. The proteins corresponding to these selected genes were considered seed nodes whose relevant scores were distributed along the edges of the PPI network. The diffusion scores were then sorted in descending order, and a top percentage was determined as a cutoff. This set of genes has a strong biological relationship with our identified genes and is further used for pathway enrichment analysis.

### 2.6. Pathway Enrichment Analysis

The pathway enrichment analysis is performed using “Metascape” [27]. This web-based tool includes functional and pathway terms from Gene Ontology (GO) processes, KEGG pathways, Reactome pathways, canonical pathways, WikiPathways, and CORUM pathways. The hypergeometric test with the Benjamini–Hochberg statistical correction algorithm is utilized. GO biological processes and KEGG pathways are chosen, with a minimum pathway size of 10 and a maximum of 500. Pathways meeting the significance criterion of a *p*-value < 0.01 are selected for further analysis.

## 3. Results

### 3.1. Potential Biomarkers by Differentially Expressed Genes and Mutual Information

In this study, we obtained fifteen formalin-fixed paraffin-embedded (FFPE) breast cancer tissues from breast cancer patients (see Section 2.2 for more details). With a low number of samples in comparison between high and low TB, traditional analysis of differentially expressed genes using DESeq2 may provide some bias due to the low data entry when calculating either a mean value or median value for comparing between the two groups of samples. Therefore, in our analysis, we propose the use of the mutual information technique (very well known as a great tool for feature selection techniques [13,28]). Since the mutual information will capture the values of all the members in one group and separate them from the other group, the final list comprises overlapping genes found to have significantly different expressions by DESeq2 and high uncertainty by the mutual information technique.

**Figure 2:**
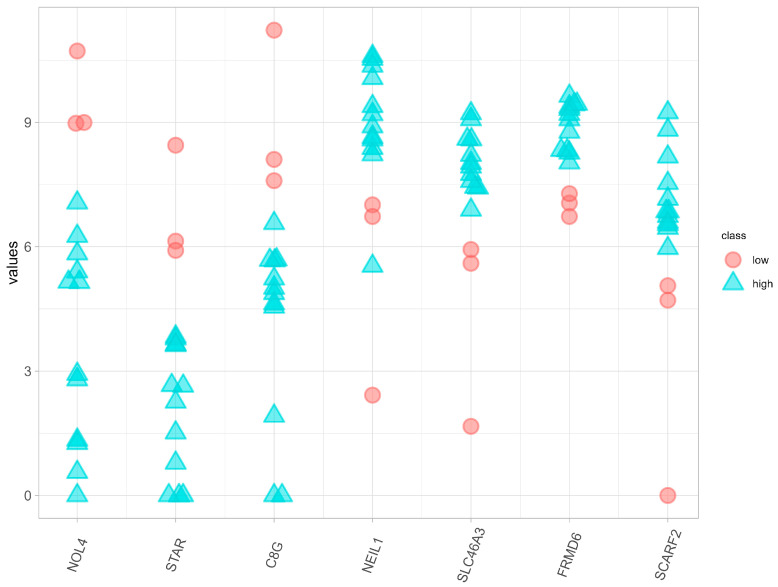
Plot depicting the expression of the identified gene set, where the *y*-axis represents the normalized counts. Note that for each gene, points with identical or closely similar values were slightly offset to enhance the visibility of individual points.

Using DESeq2, we identified 99 significantly differently expressed genes with an adjusted *p*-value <0.05 and |logFC|>1.5 (see Table S1 for the complete list). Concurrently, a higher MI score indicates a closer relationship between a gene and its class, with each gene’s MI score provided in Table S2. However, there is no standard cutoff for MI scores. The distribution of the MI scores, shown in Figure S1, indicates that only 56 genes have the highest MI score of 0.5004. Interestingly, most of these genes exhibit a high classification power, as shown in Figure S2. The results from DESeq2 revealed a significant difference in the mean of the normalized counts between the two classes, while mutual information demonstrated greater efficacy in class separation, particularly for small sample sizes. The effectiveness of mutual information in providing better classification is discussed in Section 3.2 and Section 3.3. In short, we demonstrate that the inclusion of MI or mutual information yields more reliable results and related functional pathways. Therefore, we utilized the intersection of these two approaches to enhance the likelihood of identifying potential biomarkers. Table 2 lists seven potential biomarkers identified using both methods, along with their corresponding statistical information. The baseMean values represent the average of the normalized read counts, while the log*FC* values indicate up-regulation or down-regulation. *NEIL1* exhibited the highest baseMean, indicating high expression across most samples, while *STAR* had the lowest. *NEIL1*, *SLC46A3*, *FRMD6*, and *SCARF2* were up-regulated, while *NOL4*, *STAR*, and *C8G* were down-regulated. Figure 2 depicts the normalized gene expression counts of all the biomarkers obtained.

As shown in Table 2 and Figure 2, we identified seven potential genes within breast cancer: *NOL4*, *STAR*, *C8G*, *NEIL1*, *SLC46A3*, *FRMD6*, and *SCARF2*. Their biological and tumor-related functions are summarized in Table S3. In short, *NEIL1* (Nei Like DNA Glycosylase 1) is involved in DNA repair and is up-regulated in breast and lung cancer, reducing cancer cell death and promoting cancer progression [29,30,31]. *SCARF2* (Scavenger Receptor Class F Member 2) is involved in endocytosis, lipid metabolism, and cell adhesion and is up-regulated in various cancers, promoting tumor progression [32,33]. *NOL4* (Nucleolar Protein 4) is involved in ribosome biogenesis and RNA processing and is up-regulated in various cancers, also promoting tumor progression [34,35,36,37,38]. *STAR* (Steroidogenic Acute Regulatory Protein) is crucial for steroid hormone synthesis, with its increased expression promoting certain types of breast cancer [39,40,41,42]. *C8G* (complement C8 gamma chain) facilitates membrane attack complex (MAC) formation and cell lysis and is down-regulated in breast cancer, suggesting the suppression of cell lysis [43]. *FRMD6* (FERM Domain-Containing 6, known as Willin) is an upstream regulator of the Hippo signaling pathway and is mentioned to have both tumor-suppressive and tumor-promoting effects [44,45,46,47]. *SLC46A3* (solute carrier family 46 member 3) transports folate derivatives [44]. Loss of *SLC46A3* increases the resistance of HER2+ breast cancer to trastuzumab emtansine [44,48,49,50]. Further details on these genes and the supporting literature are discussed in our discussion section.

### 3.2. Effectiveness of Potential Biomarkers in Distinguishing between Low- and High-TB Samples

**Figure 3:**
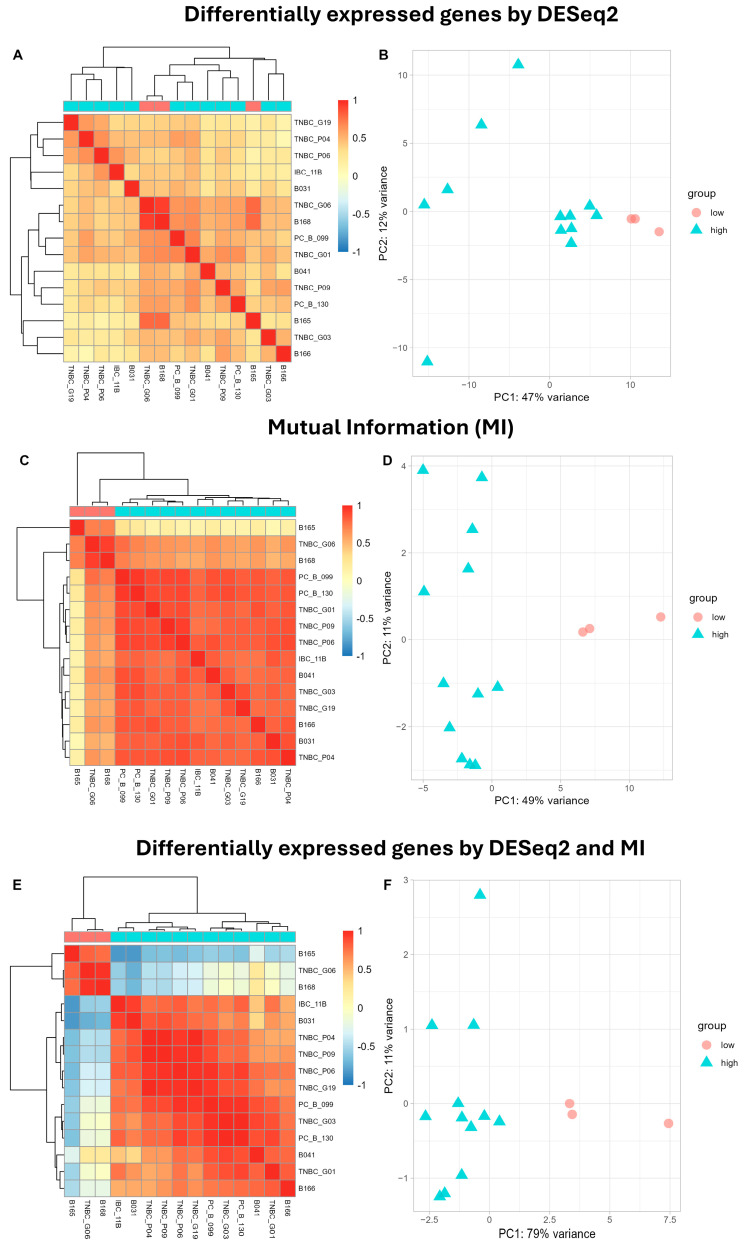
Heatmap and PCA plots utilizing the gene expression profiles of DEGs detected by DESeq2 (**A**,**B**), biomarkers detected by mutual information (**C**,**D**), and the seven potential biomarkers detected by DESeq2 and MI (**E**,**F**). The first column depicts the clusters and the correlations among expression profiles, while the second column displays the corresponding PCA plot of the first two principal components. Color intensities in the heatmaps reflect the strength of the correlation. Points in the PCA plots represent samples, with colors and shapes denoting different groups.

We assessed the effectiveness of potential biomarkers in distinguishing between low- and high-TB samples by employing heatmap plots to illustrate sample clusters based on their expression profiles. Figure 3 contains two columns. The first column illustrates the correlation between the expression profiles of the identified biomarkers for different techniques. The color intensity reflects the strength of the correlation between samples, revealing distinct expression patterns within each sample. The second column displays the corresponding principal component analysis (PCA), which visualizes the distributions of the samples based on the first two principal components derived from the expression data of the identified genes. Each sample is represented by a point, with colors and shapes indicating different TB levels. Figure 3A presents a heatmap of the correlations between the expression profiles of the 99 DEGs obtained across the BCA samples using DESeq2. Figure 3B shows a PCA plot of the first two principal components, where each point represents a sample, with the colors and shapes indicating different levels of tumor budding (TB). Notably, the three samples of low TB could not be grouped together into one cluster for the BCA samples, highlighting the limitations of solely relying on the traditional method of comparing the mean values for small sample sizes. In contrast, key biomarkers identified by the mutual information technique (Figure 3C,D) exhibit a clear distinction between the low- and high-TB samples. Finally, the heatmap and PCA plots using the expression profiles of the biomarkers detected by both DESeq2 and mutual information are shown in Figure 3E,F. These potential biomarkers can classify samples with different TB levels, a task which is not achievable using the genes identified by DESeq2 or mutual information alone. Moreover, the expression profiles of the genes discovered via the mutual information technique demonstrate improved clustering of the low- and high-TB samples compared to those of DESeq2. However, the overlapping genes found by both DESeq2 and mutual information constitute the best set for distinguishing between low- and high-TB samples.

### 3.3. Relevant Proteins and Functional Pathway Enrichment Analysis

**Figure 4:**
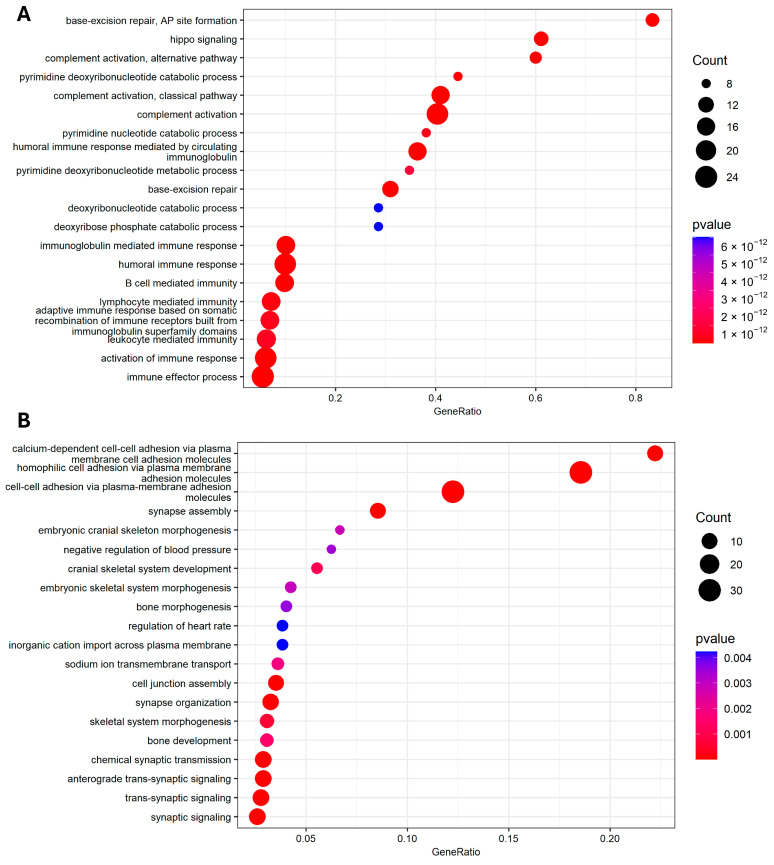
Functional enrichment for associated proteins using DESeq2 with MI (**A**) and without MI (**B**). Dot plots depicting the top 20 enriched GO biological processes. GeneRatio represents the ratio of the genes of interest appearing in the pathway to the total number of genes in the pathway.

**Figure 5:**
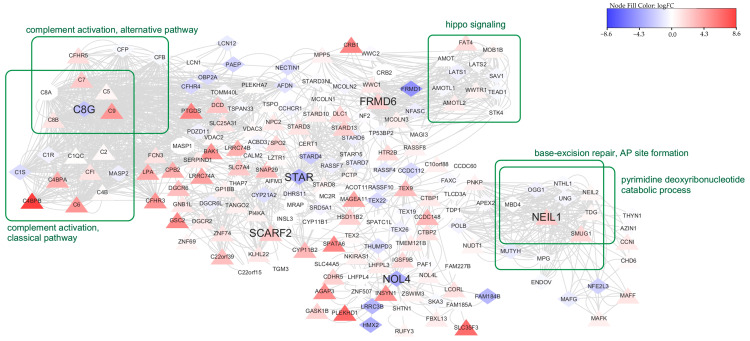
The protein–protein interaction subnetwork comprises proteins associated with the seven biomarkers detected by the diffusion technique as their diffusion scores rank above the 99th percentile. Node colors reflect the log*FC* values, with upward triangles denoting up-regulated genes, diamonds indicating down-regulated genes, and octagons representing genes with no significant change. Genes associated with a pathway of interest are grouped together.

To enhance our understanding of the functional implications associated with the seven biomarkers (*NOL4*, *STAR*, *C8G*, *NEIL1, SLC46A3*, *FRMD6*, and *SCARF2*), we employed a network diffusion technique within a human protein–protein interaction network (details in Section 2.5). By using these biomarkers as seeds, we identified an additional 191 proteins related to the seeds for pathway enrichment analysis. Enrichment analysis was conducted using “Metascape” [27], focusing on Gene Ontology (GO) biological processes and KEGG pathways, resulting in 115 significant pathways (*p*-value < 0.01), as presented in Table S4. The top 20 enriched pathways were sorted based on the gene ratio within each pathway and visualized using the “clusterProfiler” R package [51], as depicted in Figure 4A. Using Cytoscape v3.10.1 [52], we visualized the PPI subnetwork enriched with the top five pathways, showcasing their interconnectedness in Figure 5.

Figure 4A illustrates the pathway enrichment results for the biomarkers detected by both DESeq2 and the MI technique. Base excision repair AP site formation and Hippo signaling were found to be significantly enriched with high gene ratios, both exceeding 0.6. Base excision repair proteins including *XRCC1*, *APE1*, *SMUG1*, and *FEN1* were significantly associated with poor breast-cancer-specific survival [53]. Additionally, Hippo transducers play crucial roles in breast cancer formation, progression, and dissemination and are therefore suggested as potential therapeutic targets [54]. Without using MI, DEGs identified by DESeq2 served as seeds for computing the network diffusion in the PPI network. Subsequently, the top 99th percentile of the results was selected for the enrichment analysis, yielding 37 GO and KEGG pathways (Table S5).

Figure 4B presents the top 20 GO pathways sorted by gene ratio. These pathways, when compared to those identified using MI, are distinct and free of duplicates. All of the enriched pathways have very low gene ratios. The pathway with the highest gene ratio, close to 0.225, is calcium-dependent cell–cell adhesion via plasma. The absence of MI results in a variety of genes in different pathways, which consequently provides a broader seed set. Notice that this also leads to higher diffusion scores across nodes in different components, resulting in multiple top-ranked genes within the subgraphs of the PPI network, as illustrated in Figure S3. These subgraphs exhibit lower connectivity compared to the single component obtained with the seeds from DESeq2 and MI, as shown in Figure 5. Nodes that are well connected in the PPI network typically share similar functions and contribute more effectively to the enrichment analysis. Thus, incorporating MI yields a higher gene ratio (up to >0.8, as shown in Figure 4A), underscoring its significance in enhancing the analysis outcomes.

## 4. Discussion and Conclusions

In this study, we demonstrate using TempO-Seq to extract transcriptomic data on cancer cells in breast cancer subtypes to identify key biomarkers associated with high levels of TB, indicative of disease pathogenesis and progression. TempO-Seq has transformed gene expression analysis by directly measuring the activity in tissue samples without requiring RNA extraction. This technique offers exceptional precision and sensitivity [7]. Although FFPE samples typically yield fewer detected genes compared to fresh tissues, improvements have been made over time [6,55]. While RNA-Seq can provide broader gene detection and has become a standard method in many studies, it is often more prone to noise [5]. In breast cancer research, TempO-Seq was used to identify DEGs in tumors with low versus high cytoplasmic CAIX, highlighting the effective use of TempO-Seq [56].

The challenges posed by small sample sizes in cancer research are well recognized, particularly in the context of detecting differential gene expression. Traditional methods often rely on statistical tests that may lack robustness with limited sample sizes [57]. To address this, we employed mutual information techniques, which are adept at identifying features or genes differentiating two groups of small samples, such as low and high TB, as in our case. With our small sample sizes, mutual information proves more robust than traditional statistical tests, as it does not rely on assumptions about the underlying data distribution. Additionally, our study demonstrates that using mutual information is crucial for screening significant genes in network diffusion and functional enrichment analyses. While single methods like DESeq2 may detect numerous genes, including non-relevant ones, this can result in network diffusion valuing various pathways less important to high-tumor-budding breast cancer. Moreover, a larger number of seed genes in the diffusion technique can lead to diverse clusters in the PPI network, which may correspond to different functions and may be less suitable for enrichment analysis.

Combining significant differentially expressed genes (DEGs) and important genes identified by mutual information provides better potential biomarkers than either approach alone. Subsequently, we identified seven potential genes related to tumor budding in breast cancer (*NOL4*, *STAR*, *C8G*, *NEIL1*, *SLC46A3*, *FRMD6*, and *SCARF2*).

Each gene found in all breast cancer subtypes with high TB plays a different role in cellular processes. Firstly, *NEIL1* (Nei Like DNA Glycosylase 1) is involved in the DNA repair process, specifically in the base excision repair pathway, which repairs damaged DNA bases [29]. Dysregulation of the DNA repair process leads to genomic instability. Consistent with this study, previous studies showed that the expression of *NEIL1* was up-regulated in breast and lung cancer cell lines, and *NEIL1* silencing resulted in cancer cell apoptosis by increasing the pro-apoptotic protein (Bax) and decreasing the anti-apoptotic protein (Bcl-2) [30,31]. These results suggest that *NEIL1* helps maintain genomic stability, leading to a reduction in cancer cell death and eventually promoting the progression of breast and lung cancer.

*SCARF2* (Scavenger Receptor Class F Member 2) is a member of the scavenger receptor family involved in various cellular processes, including endocytosis, lipid metabolism, and cell adhesion [32]. It functions as a receptor for various ligands, including modified lipoproteins and extracellular matrix proteins [32]. Meanwhile, *NOL4* (Nucleolar Protein 4) is a nucleolar protein involved in ribosome biogenesis and RNA processing [34]. It plays a role in the assembly and maturation of ribosomes, the cellular machinery responsible for protein synthesis [34]. It was reported that *SCARF2* and *NOL4* were up-regulated in various types of cancers [33,35,36,37,38], and they promoted tumor progression and metastasis [33,35,36,37,38]. However, direct evidence linking *SCARF2* or *NOL4* to breast cancer is limited. Further research is needed to elucidate the specific mechanisms by which *SCARF2* and *NOL4* contribute to breast cancer pathogenesis.

Moreover, in this study, we found that Steroidogenic Acute Regulatory Protein (*STAR*) and complement C8 gamma chain (*C8G*) were down-regulated. *STAR* plays a crucial role in steroid hormone synthesis, particularly in transporting cholesterol into the mitochondria for steroidogenesis [39], and the increased expression of *STAR* promotes the development and progression of certain types of breast cancer, particularly hormone receptor-positive breast cancer [40,41,42]. Interestingly, *C8G* is responsible for stabilizing the interaction between the complement components C8α and C8β and facilitating their assembly into the complement membrane attack complex (MAC) [43]. Once the MAC is formed, it inserts into the membrane of the target cell, disrupting its integrity and ultimately causing cell lysis [43]. We found that in all the breast cancer subtypes with high TB, the expression of the *C8G* gene was down-regulated, suggesting that cell lysis is suppressed and may contribute to breast cancer progression. However, a previous study reported that *C8G* expression was increased in the exosome of rectal cancer patients who responded well to neoadjuvant therapy [58]. Therefore, *C8G* might have specific function in each type of cancer.

FERM Domain-Containing 6 (*FRMD6*), also known as Willin, is an upstream regulator of the Hippo signaling pathway that has recently been shown to modulate actin cytoskeleton dynamics and the mechanical phenotype of neuronal cells [44]. *FRMD6* is reported to exert both tumor-suppressive [45,46,47] and tumor-promoting [59,60] effects in various types of cancers, but still no evidence has been reported on breast cancer. However, in this study, we found that *FRMD6* gene expression was up-regulated, suggesting *FRMD6* might exhibit tumor-promoting effects in this scenario.

*SLC46A3* (solute carrier family 46 member 3) belongs to the SLC46 family of solute carriers, which are responsible for transporting folate derivatives into cells [44]. Several studies have reported the association of *SLC46A3* with breast cancer [44,48,49,50]. Loss of *SLC46A3* was reported to increase the resistance of HER2+ breast cancer to trastuzumab emtansine [44,48,49,50], and the efficacy of trastuzumab emtansine was reduced when *SLC46A3* was inhibited [44,48,49,50]. Apart from breast cancer, a low level of this gene was related to the aggressive type of hepatocellular carcinoma [61], indicating that *SLC46A3* is an important molecule that exhibits anti-tumoral effects.

Further investigation based on protein–protein interaction networks and network diffusion was performed to elucidate the specific mechanisms by which these genes contribute to breast cancer pathogenesis. This revealed enrichment in the Hippo signaling and Wnt signaling pathways, which are known to promote tumor initiation, invasion, and metastasis in several cancer types. Additionally, the use of network diffusion techniques allowed us to uncover additional proteins closely related to our identified biomarkers, thereby expanding our understanding of the functional pathways implicated in breast cancer development and progression. This integrative approach enables the identification of interconnected signaling pathways and regulatory networks that drive oncogenesis, offering new avenues for therapeutic intervention. The crucial issue with this approach is the selection of the initial seed genes or proteins in the PPI network, which iteratively distribute their scores to related partners. It is important to consider both the number of seeds and their potential significance as representatives of the specific interest, which in our case is high tumor budding in breast cancer. We demonstrated that the enrichment results, with small seed sets highly related to breast cancer, identified by DESeq2 with mutual information, provided reasonable functional relevance to cancer and more robust clusters in the PPI network.

Taken together, our study demonstrates the utility of TempO-Seq in uncovering the dynamic gene expression changes associated with breast cancer and highlights the potential of integrated analyses to elucidate the complex regulatory networks underlying cancer pathogenesis and identify novel therapeutic targets. Our findings provide insights into cancer biology and personalized treatment approaches, emphasizing the need for further investigation into the mechanisms underlying the involvement of *NOL4*, *STAR*, *C8G*, *NEIL1*, *SLC46A3*, *FRMD6*, and *SCARF2* in breast cancer pathogenesis. This presents opportunities for the development of novel therapeutic strategies and personalized treatment approaches tailored to individual patients.

In clinical data and gene expression data analysis, small sample sizes pose significant challenges that can impact the validity and reliability of the results. In our case, we have only 15 breast cancer samples (12 samples in the high-TB group vs. 3 samples in the low-TB group). To enhance the reliability of our analysis, DESeq2 and mutual information were applied, and network diffusion analysis of the PPI network was performed to extract potential biomarkers and their functional pathways. While our results provide initial insights into the gene expression changes associated with high-TB cases, the small sample size necessitates caution in the interpretation. Further experiments and analyses with larger, more comprehensive studies are required to validate these findings and explore their clinical implications.

## Figures and Tables

**Table 1:** Demographic data, tumor budding level, and clinicopathological factors of patients.

BCA Case	Age (y)	Tumor Size (cm)	OS (y)	DFS (y)	Tumor Staging	Pathological Differentiation	TB Level
B168	33	7.3	11.3	11.3	IIIB	PD	low
TNBC-G06	67	4.5	5.3	5.1	IIA	PD	low
B041	26	5.2	5.9	2.3	IIIA	PD	high
B031	50	3	4	4.1	IIIC	PD	high
B166	55	4	6.6	1.5	IIIA	PD	high
TNBC-G01	67	2.4	5.6	5.3	IIIA	MD	high
TNBC-G03	61	4	0.6	0.6	IIA	MD	high
TNBC-G19	51	4.3	5.1	5.1	IIIB	PD	high
TNBC-P04	54	3.5	3.2	3.2	IIA	PD	high
TNBC-P06	50	3	5.2	4	IIIC	MD	high
PC-B-099	50	6.5	2	1.3	IIIA	PD	high
PC-B-130	44	6	0.8	0.6	IIIC	PD	high
TNBC-P09	59	2.5	2.5	1.2	IIA	PD	high
B165	49	4	11.5	1.8	III	PD	low
IBC-11B	60	4.5	15.1	15.1	III	PD	high

OS: overall survival; y: year; DFS: disease-free survival; PD: poorly differentiated; MD: moderately differentiated.

**Table 2:** Statistical information on seven potential biomarkers.

Genes	baseMean	log*FC*	lfcSE	Stat	*p*-Value	Adjusted *p*-Value
*NOL4*	200.02	−5.14	1.1013	−4.6685	3.03 × 10^−6^	0.002490
*STAR*	36.16	−5.00	1.1492	−4.3530	1.34 × 10^−5^	0.005210
*C8G*	216.37	−4.84	0.9440	−5.1302	2.89 × 10^−7^	0.000580
*NEIL1*	588.06	3.16	0.8325	3.8001	0.000145	0.029210
*SLC46A3*	244.48	2.97	0.6544	4.5456	5.48 × 10^−6^	0.003100
*FRMD6*	441.97	2.00	0.4376	4.5627	5.05 × 10^−6^	0.003100
*SCARF2*	159.33	3.28	0.8192	4.0070	6.15 × 10^−5^	0.015180

## Data Availability

This article, along with its Supplementary Materials files, contains all the data generated or analyzed during this investigation, which are made available to the readers. Our gene expression dataset and its associated FASTQ files are publicly available in the NCBI Gene Expression Omnibus at https://www.ncbi.nlm.nih.gov/geo/ (accessed on 30 March 2024), under accession number GSE262825.

## Supplementary Materials

The following supporting information can be downloaded at https://www.mdpi.com/article/10.3390/biom14080896/s1. Table S1: Statistical information on significant DEGs; Table S2: Mutual information score for each gene [62,63,64,65,66,67,68,69,70,71,72,73,74,75,76,77,78,79,80,81,82,83,84,85,86]; Table S3: The biological and tumor-related functions of the potential biomarkers; Table S4: GO and KEGG pathway enrichment results; Table S5: GO and KEGG pathway enrichment results without using MI; Figure S1: Histogram of MI score; Figure S2: Normalized count of genes with the highest MI score; Figure S3: The PPI subnetwork containing nodes with diffusion scores rank above the 99th percentile without using MI.
