# Supplementary material for: Identification of Tumor Budding-Associated Genes in Breast Cancer through Transcriptomic Profiling and Network Diffusion Analysis
Source: Biomolecules. 2024 Jul 24;14(8):896. doi: 10.3390/biom14080896 (PMC11352152; doi:10.3390/biom14080896)
Supplement: Supplementary file 1 [file biomolecules-14-00896-s001.zip › Supplementary/TableS3 The biological and tumor-related functions of the potential biomarkers.docx]

**Table S3** The biological and tumor-related functions of the potential biomarkers.

| **Gene name/ Protein name** | **Accession no.** | **Biological function** | **Tumor-related function** |
| --- | --- | --- | --- |
| *NEIL1/* Nei-like DNA Glycosylase 1 | NC_000015.10 | a DNA repair enzyme playing a critical role in the base excision repair pathway responsible for repairing damaged DNA bases from oxidative stress or exposure to reactive oxygen species ^[1]^ | - up-regulated in estrogen receptor-positive MCF-7 breast cancer cells, however, not correlate with the outcome after radiotherapy in the TCGA breast cancer cohort ^[2]^  -silencing of NEIL1 in colorectal cell lines reduced cancer cell viability by increasing Bax and decreasing Bcl-2 leading to cancer cell apoptosis ^[3]^ |
| *SCARF2/*Scavenger Receptor Class F Member 2 | NC_000022.11 | a transmembrane protein belonging to the scavenger receptor family playing various roles in cellular processes i.e. endocytosis, lipid metabolism, cell adhesion ^[4]^ | - increased in glioblastoma, and related to poorer prognosis in glioblastoma patients ^[5]^ |
| *NOL4** /Nucleolar Protein 4 | NC_000018.10 | a protein in the nucleolus involving the formation of ribosomal subunits ^[6]^ | -overexpressed in small cell lung carcinoma ^[7-10]^  -down-regulated in patients with papillary carcinoma ^[11]^ |
| *STAR*/* Steroidogenic Acute Regulatory Protein | NC_000008.11 | a key component in the process of steroidogenesis, facilitates the transport of cholesterol from the outer to the inner mitochondrial membrane, where cholesterol is converted into pregnenolone, a precursor for the synthesis of other steroid hormones ^[12]^. | -up-regulated in breast cancer and correlated with poor overall survival ^[13-15]^. |
| *C8G*/* Complement C8 Gamma Chain | NC_000009.12 | responsible for stabilizing the interaction between complement components C8α and C8β and facilitating their assembly into the complement membrane attack complex (MAC) causing cell lysis ^[16]^. | -C8G is up-regulated in exosome isolated from rectal cancer patients who response well to neoadjuvant therapy ^[17]^. |
| *FRMD6/* FERM Domain Containing 6 | NC_000014.9 | an upstream regulator of Hippo signaling modulating actin cytoskeleton dynamics and mechanical phenotype of neuronal cells through ERK signaling, involving in neuronal differentiation, myelination, nerve injury repair, and vesicle exocytosis ^[41]^ | -inhibited glioblastoma cell proliferation, invasion *in vitro* and glioblastoma tumor growth *in vivo* ^[42]^  -down-regulated in human prostate cancer tissue and FRMD6 knockout increases prostate cancer cell viability and proliferation in *in vivo* ^[43]^  -down-regulated by HNRNPA2B1/miR-93-5p leading to enhance proliferation and metastasis in prostate cancer ^[44]^  -decreased in the clinical specimens and cell lines of thyroid cancer, increased proliferation, epithelial–mesenchymal transition, and invasion of thyroid cancer ^[45]^  -overexpressed in lung cancer tissues and associated with poor outcomes in patients with lung squamous cell carcinoma and lung adenocarcinoma ^[46]^  -high expression in colorectal cancer patients’ tissue exhibiting good prognosis and survival ^[47]^. |
| *SLC46A3/* Solute carrier family 46 member 3 | NC_000013.11 | a solute carrier family 46 member 3 encoding protein involved in the transport of molecules across cell membranes, belongs to the SLC46 family of solute carriers responsible for transporting folate derivatives into cells ^[48]^ | -loss of SLC46A3 is associated with the resistance to trastuzumab in HER2+ breast cancer ^[48,49]^  -inhibition of SLC46A3 reduced trastuzumab efficacy by inhibiting the escape of Lys-SMCC-DM1 from lysosomes into the cytoplasm ^[50,51]^.  -down-regulated in hepatocellular carcinoma patients’ tissues and related to the aggressive type ^[52]^.  -overexpressing SLC46C3 in hepatocellular carcinoma cell lines inhibited the migration and invasion as well as reduced xenograft tumor mass ^[52]^ |

*differentially down-regulated genes in high budding cases

[1] Singh, P. K. & Mistry, K. Human NEIL1 DNA glycosylase: Structure, function and polymorphisms. *Meta Gene* **11**, 49-57 (2017). <https://doi.org:https://doi.org/10.1016/j.mgene.2016.11.006>

[2] Post, A. E. M., Bussink, J., Sweep, F. & Span, P. N. Changes in DNA Damage Repair Gene Expression and Cell Cycle Gene Expression Do Not Explain Radioresistance in Tamoxifen-Resistant Breast Cancer. *Oncol Res* **28**, 33-40 (2020). <https://doi.org:10.3727/096504019x15555794826018>

[3] Xue, W. *et al.* Nei Endonuclease VIII-Like1 (NEIL1) Inhibits Apoptosis of Human Colorectal Cancer Cells. *Biomed Res Int* **2020**, 5053975 (2020). <https://doi.org:10.1155/2020/5053975>

[4] Vo, T. T. *et al.* Exploring scavenger receptor class F member 2 and the importance of scavenger receptor family in prediagnostic diseases. *Toxicol Res* **39**, 341-353 (2023). <https://doi.org:10.1007/s43188-023-00176-2>

[5] Kim, C. *et al.* Scavenger receptor class F member 2 (SCARF2) as a novel therapeutic target in glioblastoma. *Toxicol Res* **38**, 249-256 (2022). <https://doi.org:10.1007/s43188-022-00125-5>

[6] Lin, F., Zhou, J., Li, X. & Wang, X. NOL4L, a novel nuclear protein, promotes cell proliferation and metastasis by enhancing the PI3K/AKT pathway in ovarian cancer. *Biochem Biophys Res Commun* **559**, 121-128 (2021). <https://doi.org:10.1016/j.bbrc.2021.04.055>

[7] Kim, Y. R. *et al.* Cancer Testis Antigen, NOL4, Is an Immunogenic Antigen Specifically Expressed in Small-Cell Lung Cancer. *Curr Oncol* **28**, 1927-1937 (2021). <https://doi.org:10.3390/curroncol28030179>

[8] Lee, J. H. *et al.* NOL4 is a novel nuclear marker of small cell carcinoma and other neuroendocrine neoplasms. *Histol Histopathol* **37**, 1091-1098 (2022). <https://doi.org:10.14670/hh-18-540>

[9] G, P., Rathi, B. & Santoshi, S. Translational and structural vaccinomics approach to design a multi-epitope vaccine against NOL4 autologous antigen of small cell lung cancer. *Immunol Res* **71**, 909-928 (2023). <https://doi.org:10.1007/s12026-023-09404-1>

[10] Wei, Y. *et al.* Identification of Immune Subtypes and Candidate mRNA Vaccine Antigens in Small Cell Lung Cancer. *Oncologist* **28**, e1052-e1064 (2023). <https://doi.org:10.1093/oncolo/oyad193>

[11] Sheikholeslami, S. *et al.* NOL4 is Downregulated and Hyper-Methylated in Papillary Thyroid Carcinoma Suggesting Its Role as a Tumor Suppressor Gene. *Int J Endocrinol Metab* **18**, e108510 (2020). <https://doi.org:10.5812/ijem.108510>

[12] Tugaeva, K. V. & Sluchanko, N. N. Steroidogenic Acute Regulatory Protein: Structure, Functioning, and Regulation. *Biochemistry (Mosc)* **84**, S233-s253 (2019). <https://doi.org:10.1134/s0006297919140141>

[13] Manna, P. R. *et al.* Hormonal and Genetic Regulatory Events in Breast Cancer and Its Therapeutics: Importance of the Steroidogenic Acute Regulatory Protein. *Biomedicines* **10** (2022). <https://doi.org:10.3390/biomedicines10061313>

[14] Manna, P. R., Ahmed, A. U., Vartak, D., Molehin, D. & Pruitt, K. Overexpression of the steroidogenic acute regulatory protein in breast cancer: Regulation by histone deacetylase inhibition. *Biochem Biophys Res Commun* **509**, 476-482 (2019). <https://doi.org:10.1016/j.bbrc.2018.12.145>

[15] Manna, P. R. *et al.* Genomic Profiling of the Steroidogenic Acute Regulatory Protein in Breast Cancer: In Silico Assessments and a Mechanistic Perspective. *Cancers (Basel)* **11** (2019). <https://doi.org:10.3390/cancers11050623>

[16] Bayly-Jones, C., Bubeck, D. & Dunstone, M. A. The mystery behind membrane insertion: a review of the complement membrane attack complex. *Philos Trans R Soc Lond B Biol Sci* **372** (2017). <https://doi.org:10.1098/rstb.2016.0221>

[17] Strybel, U. *et al.* Molecular Composition of Serum Exosomes Could Discriminate Rectal Cancer Patients with Different Responses to Neoadjuvant Radiotherapy. *Cancers (Basel)* **14** (2022). <https://doi.org:10.3390/cancers14040993>

[18] Bayat, A. *et al.* PRICKLE2 revisited-further evidence implicating PRICKLE2 in neurodevelopmental disorders. *Eur J Hum Genet* **29**, 1235-1244 (2021). <https://doi.org:10.1038/s41431-021-00912-y>

[19] Zhu, L., Liu, Y., Tang, H. & Wang, P. Circular RNA Circ_0001777 Suppresses Lung Adenocarcinoma Progression In Vitro and In Vivo. *Biochem Genet* **61**, 704-724 (2023). <https://doi.org:10.1007/s10528-022-10284-7>

[20] Qian, H., Cui, N., Zhou, Q. & Zhang, S. Identification of miRNA biomarkers for stomach adenocarcinoma. *BMC Bioinformatics* **23**, 181 (2022). <https://doi.org:10.1186/s12859-022-04719-6>

[21] Kim, H., Ryu, J. & Lee, C. Genome-wide identification of expression quantitative trait loci for human telomerase. *Medicine (Baltimore)* **95**, e5209 (2016). <https://doi.org:10.1097/md.0000000000005209>

[22] Aloe, L., Rocco, M. L., Balzamino, B. O. & Micera, A. Nerve growth factor: role in growth, differentiation and controlling cancer cell development. *J Exp Clin Cancer Res* **35**, 116 (2016). <https://doi.org:10.1186/s13046-016-0395-y>

[23] Fernández-Nogueira, P. *et al.* Differential expression of neurogenes among breast cancer subtypes identifies high risk patients. *Oncotarget* **7**, 5313-5326 (2016). <https://doi.org:10.18632/oncotarget.6543>

[24] Chakravarthy, R., Mnich, K. & Gorman, A. M. Nerve growth factor (NGF)-mediated regulation of p75(NTR) expression contributes to chemotherapeutic resistance in triple negative breast cancer cells. *Biochem Biophys Res Commun* **478**, 1541-1547 (2016). <https://doi.org:10.1016/j.bbrc.2016.08.149>

[25] Wu, R., Li, K., Yuan, M. & Luo, K. Q. Nerve growth factor receptor increases the tumor growth and metastatic potential of triple-negative breast cancer cells. *Oncogene* **40**, 2165-2181 (2021). <https://doi.org:10.1038/s41388-021-01691-y>

[26] Bashir, N., Ishfaq, M., Mazhar, K., Khan, J. S. & Shahid, R. Upregulation of CD271 transcriptome in breast cancer promotes cell survival via NFκB pathway. *Mol Biol Rep* **49**, 487-495 (2022). <https://doi.org:10.1007/s11033-021-06900-1>

[27] Hasan, N. *et al.* LRIT3 is Required for Nyctalopin Expression and Normal ON and OFF Pathway Signaling in the Retina. *eNeuro* **7** (2020). <https://doi.org:10.1523/eneuro.0002-20.2020>

[28] Tadini-Buoninsegni, F. & Smeazzetto, S. Mechanisms of charge transfer in human copper ATPases ATP7A and ATP7B. *IUBMB Life* **69**, 218-225 (2017). <https://doi.org:10.1002/iub.1603>

[29] Shi, B., Zhang, W., Wang, T. & Cui, Z. The therapeutic and prognostic role of cuproptosis-related genes in triple negative breast cancer. *BMC Bioinformatics* **24**, 223 (2023). <https://doi.org:10.1186/s12859-023-05348-3>

[30] Zhang, D. *et al.* Comprehensive analysis of a cuproptosis-related ceRNA network implicates a potential endocrine therapy resistance mechanism in ER-positive breast cancer. *BMC Med Genomics* **16**, 96 (2023). <https://doi.org:10.1186/s12920-023-01511-0>

[31] Yu, Z., Cao, W., Ren, Y., Zhang, Q. & Liu, J. ATPase copper transporter A, negatively regulated by miR-148a-3p, contributes to cisplatin resistance in breast cancer cells. *Clin Transl Med* **10**, 57-73 (2020). <https://doi.org:10.1002/ctm2.19>

[32] Shanbhag, V. *et al.* ATP7A delivers copper to the lysyl oxidase family of enzymes and promotes tumorigenesis and metastasis. *Proc Natl Acad Sci U S A* **116**, 6836-6841 (2019). <https://doi.org:10.1073/pnas.1817473116>

[33] Hauge, H., Fjelland, K. E., Sioud, M. & Aasheim, H. C. Evidence for the involvement of FAM110C protein in cell spreading and migration. *Cell Signal* **21**, 1866-1873 (2009). <https://doi.org:10.1016/j.cellsig.2009.08.001>

[34] Li, M., Zhong, D. & Li, G. Regulatory role of local tissue signal Del-1 in cancer and inflammation: a review. *Cell Mol Biol Lett* **26**, 31 (2021). <https://doi.org:10.1186/s11658-021-00274-9>

[35] Lee, S. J. *et al.* Del-1 Expression as a Potential Biomarker in Triple-Negative Early Breast Cancer. *Oncology* **94**, 243-256 (2018). <https://doi.org:10.1159/000485658>

[36] Lee, J. E. *et al.* Identification of EDIL3 on extracellular vesicles involved in breast cancer cell invasion. *J Proteomics* **131**, 17-28 (2016). <https://doi.org:10.1016/j.jprot.2015.10.005>

[37] Moon, P. G. *et al.* Identification of Developmental Endothelial Locus-1 on Circulating Extracellular Vesicles as a Novel Biomarker for Early Breast Cancer Detection. *Clin Cancer Res* **22**, 1757-1766 (2016). <https://doi.org:10.1158/1078-0432.Ccr-15-0654>

[38] Lee, S. J. *et al.* Exosomal Del-1 as a Potent Diagnostic Marker for Breast Cancer: Prospective Cohort Study. *Clin Breast Cancer* **21**, e748-e756 (2021). <https://doi.org:10.1016/j.clbc.2021.02.002>

[39] Lee, J. *et al.* Overcoming Tamoxifen Resistance by Regulation of Del-1 in Breast Cancer. *Oncology* **97**, 180-188 (2019). <https://doi.org:10.1159/000501340>

[40] Nommick, A. *et al.* Lrrcc1 and Ccdc61 are conserved effectors of multiciliated cell function. *J Cell Sci* **135** (2022). <https://doi.org:10.1242/jcs.258960>

[41] Chen, D., Yu, W., Aitken, L. & Gunn-Moore, F. Willin/FRMD6: A Multi-Functional Neuronal Protein Associated with Alzheimer's Disease. *Cells* **10** (2021). <https://doi.org:10.3390/cells10113024>

[42] Xu, Y., Wang, K. & Yu, Q. FRMD6 inhibits human glioblastoma growth and progression by negatively regulating activity of receptor tyrosine kinases. *Oncotarget* **7**, 70080-70091 (2016). <https://doi.org:10.18632/oncotarget.12148>

[43] Haldrup, J. *et al.* FRMD6 has tumor suppressor functions in prostate cancer. *Oncogene* **40**, 763-776 (2021). <https://doi.org:10.1038/s41388-020-01548-w>

[44] Sun, M. *et al.* Activation of the HNRNPA2B1/miR-93-5p/FRMD6 axis facilitates prostate cancer progression in an m6A-dependent manner. *J Cancer* **14**, 1242-1256 (2023). <https://doi.org:10.7150/jca.83863>

[45] Wang, W. *et al.* FERM domain-containing protein 6 exerts a tumor-inhibiting role in thyroid cancer by antagonizing oncogenic YAP1. *Biofactors* **48**, 428-441 (2022). <https://doi.org:10.1002/biof.1791>

[46] Wang, T. *et al.* FERM domain-containing protein FRMD6 activates the mTOR signaling pathway and promotes lung cancer progression. *Front Med* **17**, 714-728 (2023). <https://doi.org:10.1007/s11684-022-0959-5>

[47] von Koskull, A., Hagström, J., Haglund, C., Kaprio, T. & Böckelman, C. High-tissue FRMD6 expression predicts better outcomes among colorectal cancer patients. *Biomarkers*, 1-7 (2024). <https://doi.org:10.1080/1354750x.2024.2321916>

[48] Pegram, M. D., Miles, D., Tsui, C. K. & Zong, Y. HER2-Overexpressing/Amplified Breast Cancer as a Testing Ground for Antibody-Drug Conjugate Drug Development in Solid Tumors. *Clin Cancer Res* **26**, 775-786 (2020). <https://doi.org:10.1158/1078-0432.Ccr-18-1976>

[49] Li, G. *et al.* Mechanisms of Acquired Resistance to Trastuzumab Emtansine in Breast Cancer Cells. *Mol Cancer Ther* **17**, 1441-1453 (2018). <https://doi.org:10.1158/1535-7163.Mct-17-0296>

[50] Tomabechi, R. *et al.* SLC46A3 is a lysosomal proton-coupled steroid conjugate and bile acid transporter involved in transport of active catabolites of T-DM1. *PNAS Nexus* **1**, pgac063 (2022). <https://doi.org:10.1093/pnasnexus/pgac063>

[51] Kiyomiya, K. *et al.* Macrolide and Ketolide Antibiotics Inhibit the Cytotoxic Effect of Trastuzumab Emtansine in HER2-Positive Breast Cancer Cells: Implication of a Potential Drug-ADC Interaction in Cancer Chemotherapy. *Mol Pharm* **20**, 6130-6139 (2023). <https://doi.org:10.1021/acs.molpharmaceut.3c00490>

[52] Zhao, Q. *et al.* Increased expression of SLC46A3 to oppose the progression of hepatocellular carcinoma and its effect on sorafenib therapy. *Biomed Pharmacother* **114**, 108864 (2019). <https://doi.org:10.1016/j.biopha.2019.108864>
