# Supplementary figures and images for: Identification of Tumor Budding-Associated Genes in Breast Cancer through Transcriptomic Profiling and Network Diffusion Analysis
Source: Biomolecules. 2024 Jul 24;14(8):896. doi: 10.3390/biom14080896 (PMC11352152; doi:10.3390/biom14080896)

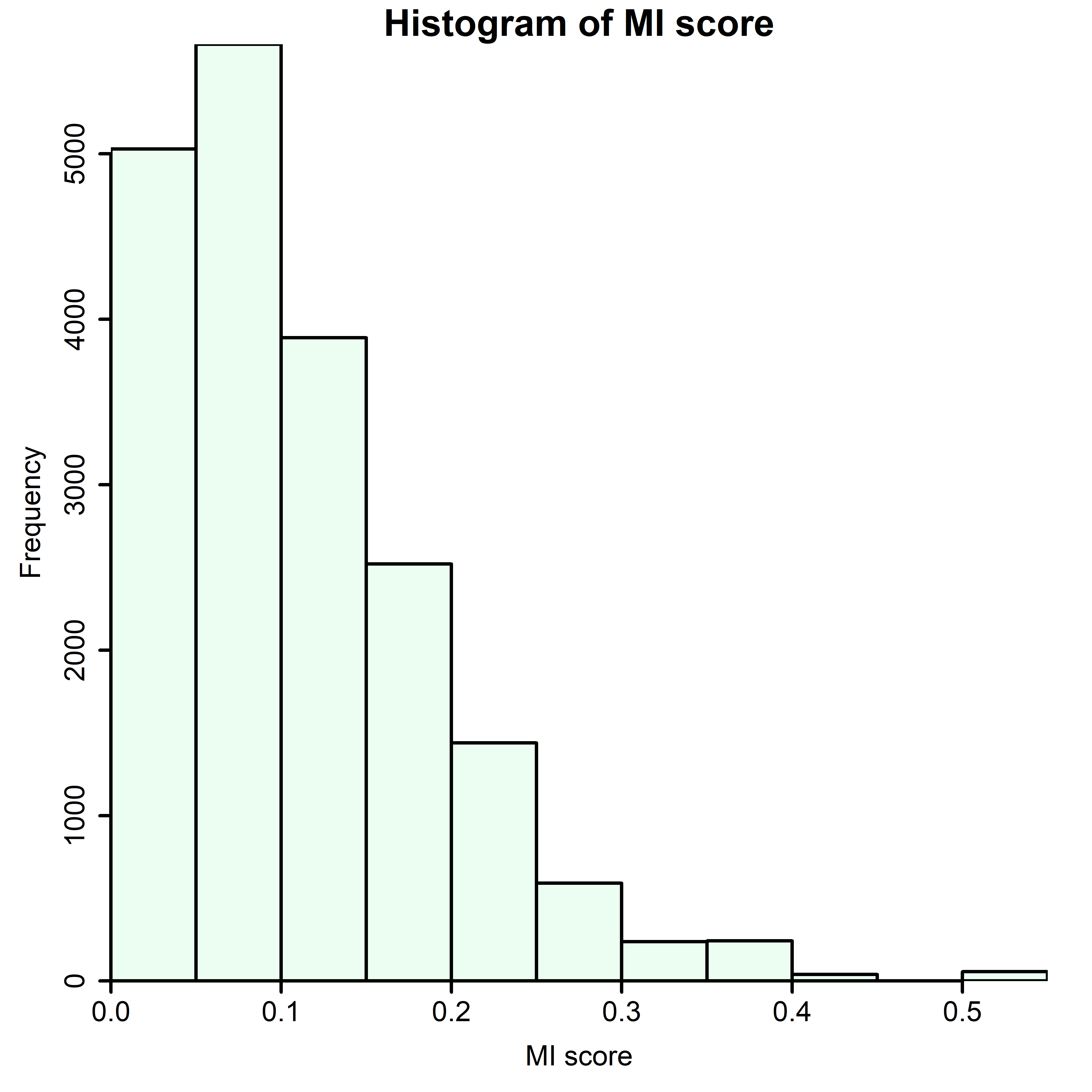

Supplement: Supplementary file 1 [file biomolecules-14-00896-s001.zip › Supplementary/Figure S1 Histogram of MI score.png]

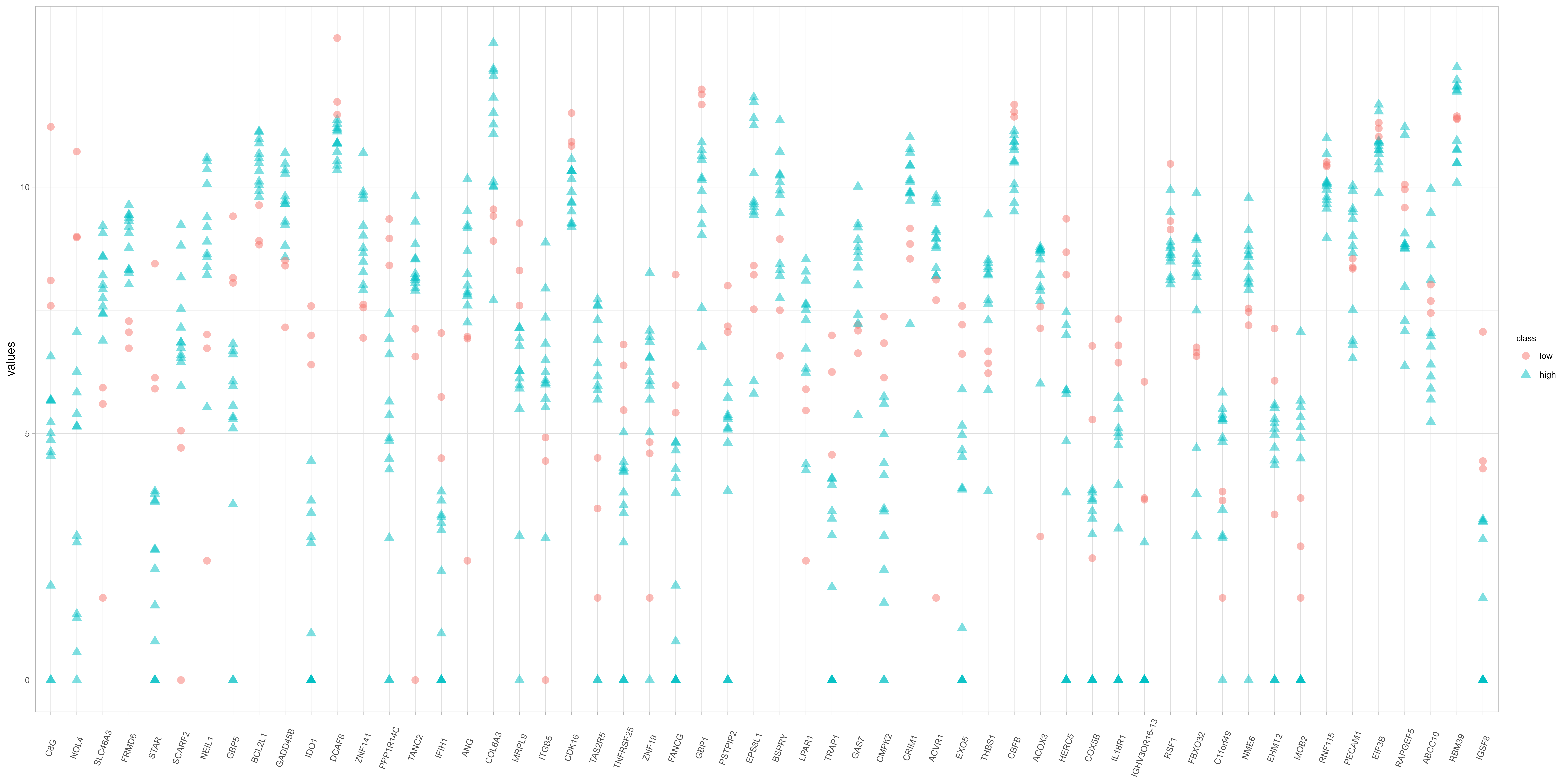

Supplement: Supplementary file 1 [file biomolecules-14-00896-s001.zip › Supplementary/Figure S2 Normalized count of top MI genes.png]

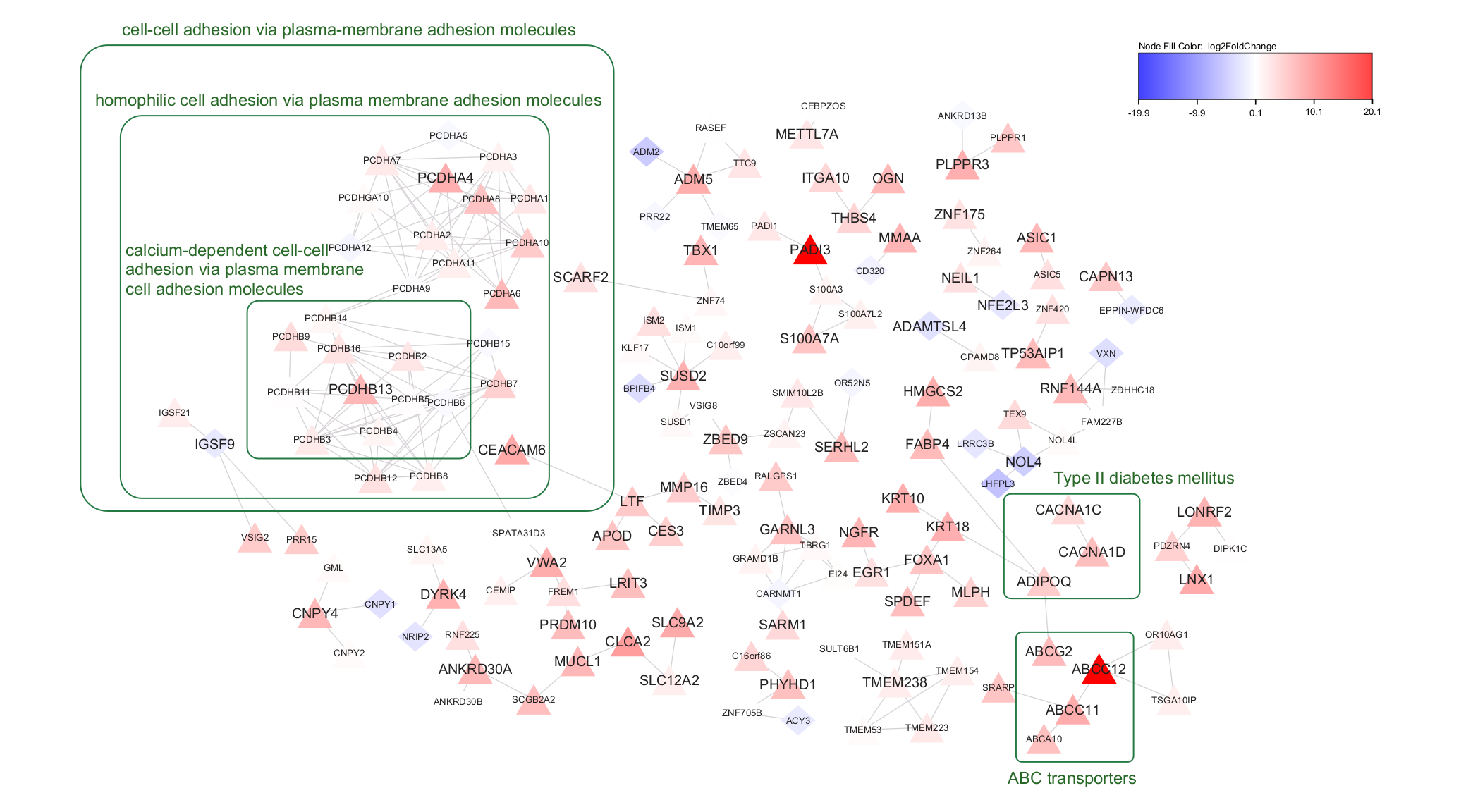

Supplement: Supplementary file 1 [file biomolecules-14-00896-s001.zip › Supplementary/Figure S3 PPI result (without MI).png]
